# *catena*-Poly[[diaqua­cadmium(II)]-μ-4,4′-sulfonyl­dibenzoato-κ^2^ *O* ^1^:*O* ^1′^]

**DOI:** 10.1107/S1600536810039711

**Published:** 2010-10-13

**Authors:** Chang-mei Jiao

**Affiliations:** a Department of Chemistry, Yancheng Teachers’ College, Yancheng 224002, People’s Republic of China

## Abstract

The title compound, [Cd(C_14_H_8_O_6_S)(H_2_O)_2_]_*n*_, comprises zigzag chains parallel to [111] of alternating [Cd(H_2_O)_2_]^2+^ and sulfonyl­dibenzoate units, with the Cd and S atoms lying on crystallographic twofold axes. The central Cd^II^ ion is in a slightly distorted octa­hedral geometry, coordinated by six O atoms from two carboxyl­ate groups and two water O atoms. An intra­molecular C—H⋯O hydrogen bond occurs. In the crystal, inter­molecular hydrogen bonds between carboxyl­ate O atoms and coordinated water mol­ecules in adjacent chains lead to the formation of a three-dimensional network structure. The compound is isotypic with the Zn analog.

## Related literature

For related compounds based on 4,4′-sulfonyl­dibenzoic acid, see: Xiao *et al.* (2007 ▶); Wu *et al.* (2007 ▶); Miyazawa *et al.* (2009 ▶); Wang *et al.* (2009 ▶). For the isotypic Zn analog, see: Pan *et al.* (2007 ▶). For potential application of metal-organic frameworks, see: Eddaoudi *et al.* (2001 ▶); Ferey *et al.* (2005 ▶); Kitagawa *et al.* (2004 ▶).
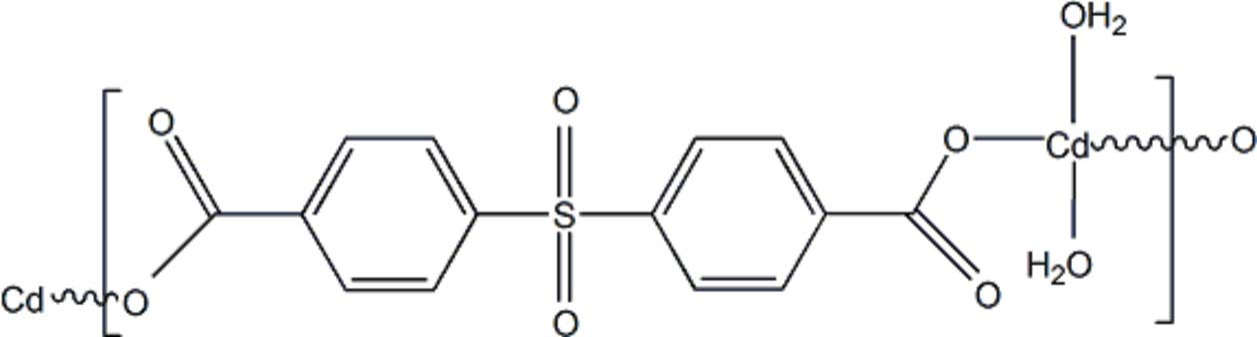


## Experimental

### 

#### Crystal data

[Cd(C_14_H_8_O_6_S)(H_2_O)_2_]
*M* *_r_* = 452.72
Monoclinic, 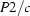

*a* = 13.293 (3) Å
*b* = 5.2742 (12) Å
*c* = 12.156 (3) Å
β = 116.145 (2)°
*V* = 765.1 (3) Å^3^
*Z* = 2
Mo *K*α radiation
μ = 1.61 mm^−1^
*T* = 298 K
0.21 × 0.19 × 0.15 mm

#### Data collection

Bruker SMART CCD area-detector diffractometer
Absorption correction: multi-scan (*SADABS*; Bruker, 2000 ▶) *T*_min_ = 0.721, *T*_max_ = 0.786
3574 measured reflections
1364 independent reflections
1325 reflections with *I* > 2σ(*I*)
*R*_int_ = 0.073

#### Refinement

*R*[*F* ^2^ > 2σ(*F* ^2^)] = 0.032
*wR*(*F* ^2^) = 0.102
*S* = 1.24
1361 reflections
110 parameters
3 restraints
H-atom parameters constrained
Δρ_max_ = 0.82 e Å^−3^
Δρ_min_ = −1.00 e Å^−3^

### 

Data collection: *SMART* (Bruker, 2000 ▶); cell refinement: *SAINT* (Bruker, 2000 ▶); data reduction: *SAINT*; program(s) used to solve structure: *SHELXS97* (Sheldrick, 2008 ▶); program(s) used to refine structure: *SHELXL97* (Sheldrick, 2008 ▶); molecular graphics: *SHELXTL* (Sheldrick, 2008 ▶) and *DIAMOND* (Brandenburg, 1999 ▶); software used to prepare material for publication: *SHELXTL*.

## Supplementary Material

Crystal structure: contains datablocks I, global. DOI: 10.1107/S1600536810039711/bx2311sup1.cif

Structure factors: contains datablocks I. DOI: 10.1107/S1600536810039711/bx2311Isup2.hkl

Additional supplementary materials:  crystallographic information; 3D view; checkCIF report

## Figures and Tables

**Table 1:** Hydrogen-bond geometry (Å, °)

*D*—H⋯*A*	*D*—H	H⋯*A*	*D*⋯*A*	*D*—H⋯*A*
O4—H4*B*⋯O1^i^	0.85	1.97	2.7479 (14)	151
O4—H4*A*⋯O2^ii^	0.85	2.00	2.7364 (15)	145
C6—H6⋯O3	0.93	2.55	2.9208 (16)	104

Symmetry codes: (i) 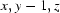; (ii) 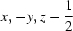.

**Fig. 1.**
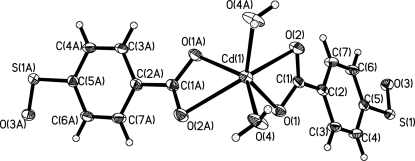
: The structure of (I), showing the atom-numbering scheme. Displacement ellipsoids are plotted at the 30% probability level. [Symmetry codes: (A) –x, y, 3/2 - z]

**Fig. 2.**
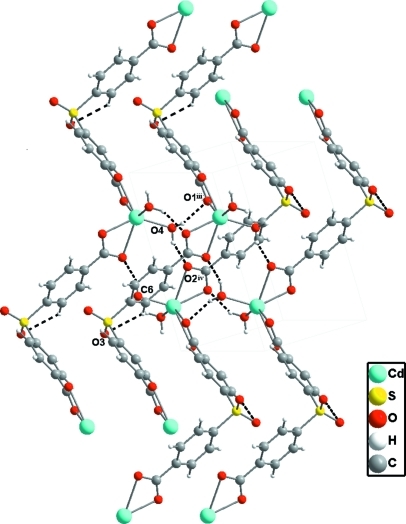
: A view of (I), showing the three-dimensional framework constructed via O—H···O hydrogen bonds. Hydrogen bonds are depicted as dashed lines. [Symmetry codes: (iii) x, y - 1, z; (iv) x, -y, -1/2 + z]

## supplementary crystallographic information

### Comment

In recent years, much attention has been focused on the construction of metal organic frameworks (MOFs) not only because of their fascinating structures and topologies but also owing to their potential application in many fields such as magnetism, catalysis, nonlinear optics. (Eddaoudi, *et al.*, 2001; Kitagawa *et al.*, 2004; Ferey *et al.*, 2005.). The main method to construct such complexes is to use multidentate organic ligands. The organic aromatic polycarboxylate ligands are an important family of multidentate ligands. The 4,4'-sulfonyldibenzoic acid has been widely used in the construction of metal organic frameworks because of two carboxylate functions and its structural flexibility.(Xiao *et al.*, 2007; Wu *et al.*, 2007; Miyazawa *et al.*, 2009; Wang *et al.*, 2009.) We report here the synthesis and crystal structure of the title compound (I) based on 4,4'-sulfonyldibenzoic acid which is isostructural to the reported compound by Pan *et al.*, 2007.

As shown in Fig. 1, the Cd centres in (I) are six-coordinate in a highly distorted octahedral geometry, involving four O atom donors of two 4,4'-sulfonyldibenzoic acid ligands and two coordinated water molecules, while the carboxylate group of 4,4'-sulfonyldibenzoic acid adopts µ_2_-η^1^:η^1^– chelating mode in this structure. The structure of (I) comprises *zigzag* chains of alternating [Cd(H_2_O)_2_]^2+^ and sulfonyldibenzoate unit, with their respective Cd and S atoms lying on crystallographic twofold axes. In the crystal structure there are three hydrogen bonds, two O—H···O intermolecular and one C—H···O intramolecular interactions, lead to the formation of a three dimensional network structure. Fig 2, Table 1.

### Experimental

The title compound, (I), was prepared by the hydrothermal reaction of Cd(NO_3_)_2_.6H_2_O (34.5 mg, 0.1 mmol), 4,4'-sulfonyldibenzoic (30 mg, 0.1 mmol), 1,4-Bis(1,2,4-triazol-1-yl)butane (19.2 mg, 0.1 mmol), and NaOH (8.0 mg, 0.2 mmol) in H_2_O (10 ml) was sealed in a 16 ml Teflon-lined stainless steel container and heated at 180 °C for 72 h. After cooling to room temperature, block colorless crystals of (I) were collected by filtration and washed by water and ethanol several times. (yield 47.25%, based on Cd). Elemental analysis for C_14_H_12_CdO_8_S (Mr = 452.72): C 37.14, H 2.67; found: 43.61, H 2.69.

### Refinement

H atoms bonded to coordinated water oxygen atom were located in a difference Fourier map and fixed in the refinement, with *U*_iso_(H)=1.2*U*_eq_(O). All C-bound H atoms were positioned in calculated positions and refined using a riding model, with C—H = 0.93?(aromatic) and *U*_iso_(H) = 1.2*U*_eq_(C).

### Crystal data

**Table:**

[Cd(C_14_H_8_O_6_S)(H_2_O)_2_]	*F*(000) = 448
*M**_r_* = 452.72	*D*_x_ = 1.965 Mg m^−^^3^
Monoclinic, *P*2/*c*	Mo *K*α radiation, λ = 0.71073 Å
Hall symbol: -P 2yc	Cell parameters from 4318 reflections
*a* = 13.293 (3) Å	θ = 2.2–27.2°
*b* = 5.2742 (12) Å	µ = 1.61 mm^−^^1^
*c* = 12.156 (3) Å	*T* = 298 K
β = 116.145 (2)°	Block, white
*V* = 765.1 (3) Å^3^	0.21 × 0.19 × 0.15 mm
*Z* = 2	

### Data collection

**Table:**

Bruker SMART CCD area-detector diffractometer	1364 independent reflections
Radiation source: fine-focus sealed tube	1325 reflections with *I* > 2σ(*I*)
graphite	*R*_int_ = 0.073
phi and ω scans	θ_max_ = 25.1°, θ_min_ = 1.7°
Absorption correction: multi-scan (*SADABS*; Bruker, 2000)	*h* = −8→15
*T*_min_ = 0.721, *T*_max_ = 0.786	*k* = −6→6
3574 measured reflections	*l* = −14→12

### Refinement

**Table:**

Refinement on *F*^2^	Primary atom site location: structure-invariant direct methods
Least-squares matrix: full	Secondary atom site location: difference Fourier map
*R*[*F*^2^ > 2σ(*F*^2^)] = 0.032	Hydrogen site location: inferred from neighbouring sites
*wR*(*F*^2^) = 0.102	H-atom parameters constrained
*S* = 1.24	*w* = 1/[σ^2^(*F*_o_^2^) + (0.0652*P*)^2^] where *P* = (*F*_o_^2^ + 2*F*_c_^2^)/3
1361 reflections	(Δ/σ)_max_ = 0.004
110 parameters	Δρ_max_ = 0.82 e Å^−^^3^
3 restraints	Δρ_min_ = −1.00 e Å^−^^3^

### Special details

**Table:**

Geometry. All e.s.d.'s (except the e.s.d. in the dihedral angle between two l.s. planes) are estimated using the full covariance matrix. The cell e.s.d.'s are taken into account individually in the estimation of e.s.d.'s in distances, angles and torsion angles; correlations between e.s.d.'s in cell parameters are only used when they are defined by crystal symmetry. An approximate (isotropic) treatment of cell e.s.d.'s is used for estimating e.s.d.'s involving l.s. planes.
Refinement. Refinement of *F*^2^ against ALL reflections. The weighted *R*-factor *wR* and goodness of fit *S* are based on *F*^2^, conventional *R*-factors *R* are based on *F*, with *F* set to zero for negative *F*^2^. The threshold expression of *F*^2^ > σ(*F*^2^) is used only for calculating *R*-factors(gt) *etc*. and is not relevant to the choice of reflections for refinement. *R*-factors based on *F*^2^ are statistically about twice as large as those based on *F*, and *R*- factors based on ALL data will be even larger.

### Fractional atomic coordinates and isotropic or equivalent isotropic displacement parameters (Å^2^)

**Table:**

	*x*	*y*	*z*	*U*_iso_*/*U*_eq_	
Cd1	0.0000	0.06452 (2)	0.7500	0.03071 (4)	
S1	0.5000	1.11217 (9)	1.2500	0.02880 (11)	
O1	0.14145 (7)	0.34261 (18)	0.81726 (7)	0.0355 (2)	
O2	0.08657 (7)	0.25919 (19)	0.95927 (7)	0.0382 (2)	
O3	0.45660 (6)	1.24838 (18)	1.32215 (7)	0.0379 (2)	
O4	0.07556 (8)	−0.2236 (2)	0.67828 (7)	0.0504 (3)	
H4A	0.0490	−0.2211	0.6006	0.060*	
H4B	0.0724	−0.3725	0.7034	0.060*	
C1	0.14914 (9)	0.3776 (3)	0.92426 (9)	0.0286 (2)	
C2	0.23354 (12)	0.5638 (2)	1.00469 (11)	0.0304 (4)	
C3	0.32577 (10)	0.6180 (3)	0.98250 (11)	0.0354 (3)	
H3	0.3327	0.5418	0.9172	0.043*	
C4	0.40667 (9)	0.7851 (3)	1.05787 (10)	0.0365 (3)	
H4	0.4693	0.8180	1.0449	0.044*	
C5	0.39451 (10)	0.9035 (2)	1.15258 (11)	0.0290 (3)	
C6	0.30158 (9)	0.8548 (3)	1.17385 (10)	0.0351 (3)	
H6	0.2937	0.9353	1.2377	0.042*	
C7	0.22143 (9)	0.6857 (3)	1.09904 (10)	0.0349 (3)	
H7	0.1588	0.6533	1.1121	0.042*	

### Atomic displacement parameters (Å^2^)

**Table:**

	*U*^11^	*U*^22^	*U*^33^	*U*^12^	*U*^13^	*U*^23^
Cd1	0.03760 (6)	0.02817 (8)	0.02969 (5)	0.000	0.01787 (4)	0.000
S1	0.03045 (16)	0.03051 (18)	0.02660 (15)	0.000	0.01361 (12)	0.000
O1	0.0430 (3)	0.0402 (4)	0.0285 (2)	−0.0069 (3)	0.0206 (2)	−0.0069 (3)
O2	0.0473 (3)	0.0419 (5)	0.0299 (3)	−0.0117 (3)	0.0211 (2)	−0.0022 (3)
O3	0.0406 (3)	0.0381 (5)	0.0387 (4)	0.0037 (4)	0.0208 (3)	−0.0066 (3)
O4	0.0902 (5)	0.0382 (5)	0.0411 (3)	0.0184 (4)	0.0456 (3)	0.0079 (4)
C1	0.0348 (4)	0.0289 (5)	0.0234 (3)	0.0038 (4)	0.0140 (2)	0.0057 (4)
C2	0.0345 (5)	0.0342 (8)	0.0240 (5)	0.0000 (4)	0.0143 (4)	0.0034 (4)
C3	0.0375 (5)	0.0461 (6)	0.0309 (4)	−0.0039 (5)	0.0226 (3)	−0.0076 (5)
C4	0.0348 (4)	0.0464 (7)	0.0378 (4)	−0.0055 (5)	0.0248 (3)	−0.0056 (5)
C5	0.0305 (5)	0.0305 (6)	0.0253 (5)	0.0004 (4)	0.0116 (4)	0.0028 (4)
C6	0.0394 (5)	0.0426 (6)	0.0314 (4)	−0.0039 (6)	0.0231 (3)	−0.0065 (5)
C7	0.0375 (4)	0.0405 (7)	0.0364 (4)	−0.0060 (5)	0.0252 (3)	−0.0035 (5)

### Geometric parameters (Å, °)

**Table:**

Cd1—O4	2.2023 (11)	O4—H4A	0.8500
Cd1—O4^i^	2.2023 (11)	O4—H4B	0.8499
Cd1—O1	2.2362 (10)	C1—C2	1.4871 (16)
Cd1—O1^i^	2.2362 (9)	C2—C7	1.385 (2)
Cd1—O2	2.5040 (9)	C2—C3	1.396 (2)
Cd1—O2^i^	2.5040 (10)	C3—C4	1.3801 (18)
Cd1—C1^i^	2.7283 (12)	C3—H3	0.9300
S1—O3	1.4367 (10)	C4—C5	1.380 (2)
S1—O3^ii^	1.4367 (10)	C4—H4	0.9300
S1—C5	1.7655 (12)	C5—C6	1.393 (2)
S1—C5^ii^	1.7655 (12)	C6—C7	1.3798 (17)
O1—C1	1.2725 (15)	C6—H6	0.9300
O2—C1	1.2550 (17)	C7—H7	0.9300
			
O4—Cd1—O4^i^	92.73 (6)	C1—O1—Cd1	98.32 (8)
O4—Cd1—O1	98.09 (4)	C1—O2—Cd1	86.34 (7)
O4^i^—Cd1—O1	139.87 (3)	Cd1—O4—H4A	113.0
O4—Cd1—O1^i^	139.87 (3)	Cd1—O4—H4B	113.1
O4^i^—Cd1—O1^i^	98.09 (4)	H4A—O4—H4B	110.5
O1—Cd1—O1^i^	98.02 (5)	O2—C1—O1	120.51 (10)
O4—Cd1—O2	126.84 (3)	O2—C1—C2	121.76 (11)
O4^i^—Cd1—O2	88.07 (4)	O1—C1—C2	117.73 (12)
O1—Cd1—O2	54.80 (3)	C7—C2—C3	119.83 (11)
O1^i^—Cd1—O2	92.21 (3)	C7—C2—C1	121.45 (14)
O4—Cd1—O2^i^	88.07 (4)	C3—C2—C1	118.72 (13)
O4^i^—Cd1—O2^i^	126.84 (3)	C4—C3—C2	119.75 (13)
O1—Cd1—O2^i^	92.21 (3)	C4—C3—H3	120.1
O1^i^—Cd1—O2^i^	54.80 (3)	C2—C3—H3	120.1
O2—Cd1—O2^i^	131.59 (5)	C5—C4—C3	119.95 (13)
O4—Cd1—C1^i^	114.36 (4)	C5—C4—H4	120.0
O4^i^—Cd1—C1^i^	115.02 (4)	C3—C4—H4	120.0
O1—Cd1—C1^i^	95.34 (4)	C4—C5—C6	120.73 (11)
O1^i^—Cd1—C1^i^	27.48 (4)	C4—C5—S1	119.41 (11)
O2—Cd1—C1^i^	113.08 (4)	C6—C5—S1	119.84 (10)
O2^i^—Cd1—C1^i^	27.33 (4)	C7—C6—C5	119.16 (12)
O3—S1—O3^ii^	119.99 (9)	C7—C6—H6	120.4
O3—S1—C5	107.90 (6)	C5—C6—H6	120.4
O3^ii^—S1—C5	108.43 (6)	C6—C7—C2	120.54 (13)
O3—S1—C5^ii^	108.43 (6)	C6—C7—H7	119.7
O3^ii^—S1—C5^ii^	107.90 (6)	C2—C7—H7	119.7
C5—S1—C5^ii^	102.86 (8)		
			
O4—Cd1—O1—C1	−130.73 (7)	O2—C1—C2—C3	156.57 (12)
O4^i^—Cd1—O1—C1	−26.66 (10)	O1—C1—C2—C3	−23.92 (16)
O1^i^—Cd1—O1—C1	86.14 (8)	C7—C2—C3—C4	2.62 (18)
O2—Cd1—O1—C1	−0.88 (7)	C1—C2—C3—C4	−178.34 (11)
O2^i^—Cd1—O1—C1	140.90 (7)	C2—C3—C4—C5	−1.87 (19)
C1^i^—Cd1—O1—C1	113.69 (8)	C3—C4—C5—C6	0.49 (19)
O4—Cd1—O2—C1	72.63 (8)	C3—C4—C5—S1	178.80 (10)
O4^i^—Cd1—O2—C1	164.60 (7)	O3^ii^—S1—C5—C4	36.48 (11)
O1^i^—Cd1—O2—C1	−97.38 (8)	O3—S1—C5—C4	167.88 (10)
O1—Cd1—O2—C1	0.89 (7)	C5^ii^—S1—C5—C4	−77.63 (10)
O2^i^—Cd1—O2—C1	−54.85 (7)	O3^ii^—S1—C5—C6	−145.19 (10)
C1^i^—Cd1—O2—C1	−78.93 (9)	O3—S1—C5—C6	−13.78 (12)
Cd1—O2—C1—O1	−1.48 (11)	C5^ii^—S1—C5—C6	100.71 (11)
Cd1—O2—C1—C2	178.02 (11)	C4—C5—C6—C7	0.15 (19)
Cd1—O1—C1—O2	1.67 (13)	S1—C5—C6—C7	−178.16 (10)
Cd1—O1—C1—C2	−177.85 (9)	C5—C6—C7—C2	0.61 (18)
O2—C1—C2—C7	−24.41 (18)	C3—C2—C7—C6	−2.00 (18)
O1—C1—C2—C7	155.11 (11)	C1—C2—C7—C6	178.99 (11)

Symmetry codes: (i) −*x*, *y*, −*z*+3/2; (ii) −*x*+1, *y*, −*z*+5/2.

### Hydrogen-bond geometry (Å, °)

**Table:**

*D*—H···*A*	*D*—H	H···*A*	*D*···*A*	*D*—H···*A*
O4—H4B···O1^iii^	0.85	1.97	2.7479 (14)	151
O4—H4A···O2^iv^	0.85	2.00	2.7364 (15)	145
C6—H6···O3	0.93	2.55	2.9208 (16)	104

Symmetry codes: (iii) *x*, *y*−1, *z*; (iv) *x*, −*y*, *z*−1/2.
